# *Cis*-eQTLs in seven duck tissues identify novel candidate genes for growth and carcass traits

**DOI:** 10.1186/s12864-024-10338-7

**Published:** 2024-04-30

**Authors:** Wentao Cai, Jian Hu, Yunsheng Zhang, Zhanbao Guo, Zhengkui Zhou, Shuisheng Hou

**Affiliations:** Institute of Animal Science, Chinese Academy of Agricultural Sciences, Beijing, 100193 China

**Keywords:** eQTLs, Duck, GWAS, Colocalization, Growth, Carcass traits

## Abstract

**Background:**

Expression quantitative trait loci (eQTL) studies aim to understand the influence of genetic variants on gene expression. The colocalization of eQTL mapping and GWAS strategy could help identify essential candidate genes and causal DNA variants vital to complex traits in human and many farm animals. However, eQTL mapping has not been conducted in ducks. It is desirable to know whether eQTLs within GWAS signals contributed to duck economic traits.

**Results:**

In this study, we conducted an eQTL analysis using publicly available RNA sequencing data from 820 samples, focusing on liver, muscle, blood, adipose, ovary, spleen, and lung tissues. We identified 113,374 *cis*-eQTLs for 12,266 genes, a substantial fraction 39.1% of which were discovered in at least two tissues. The *cis*-eQTLs of blood were less conserved across tissues, while *cis*-eQTLs from any tissue exhibit a strong sharing pattern to liver tissue. Colocalization between *cis*-eQTLs and genome-wide association studies (GWAS) of 50 traits uncovered new associations between gene expression and potential loci influencing growth and carcass traits. *SRSF4*, *GSS,* and *IGF2BP1* in liver, *NDUFC2* in muscle, *ELF3* in adipose, and *RUNDC1* in blood could serve as the candidate genes for duck growth and carcass traits.

**Conclusions:**

Our findings highlight substantial differences in genetic regulation of gene expression across duck primary tissues, shedding light on potential mechanisms through which candidate genes may impact growth and carcass traits. Furthermore, this availability of eQTL data offers a valuable resource for deciphering further genetic association signals that may arise from ongoing extensive endeavors aimed at enhancing duck production traits.

**Supplementary Information:**

The online version contains supplementary material available at 10.1186/s12864-024-10338-7.

## Background

Ducks are frequently raised as poultry for the consumption of their meat and eggs by humans. Duck meat is commonly acknowledged for its rich flavor, high amino acid and polyunsaturated fatty acid content, and comparatively low-fat levels [1, 2]. Breeders consider traits related to growth and body weight composition to be the most critical in broiler ducks [3]. Meat production and growth traits are influenced by numerous polygenic QTLs, with each QTL making a small contribution to the trait [4, 5]. To overcome the challenge, genome-wide associated studies (GWAS) have been widely used to identify genetic loci affecting complex traits such as growth rate and meat production. Currently, several candidate loci associated with economic traits have been identified [6, 7]. For example, the variants of *IGF2BP1* determined the variation in body size and feed efficiency [8]. However, how to mechanistically decipher these candidate loci contributing to agronomic traits in ducks is a challenge. Nowadays, with the development of molecular phenotypic measurements, we can elucidate the genetic architecture of complex traits using these molecular phenotypes, such as gene expression information [9, 10].

Analyzing expression quantitative trait loci (eQTLs) is the most effective approach for evaluating how sequence variants influence gene expression within their native genomic and cellular environments [11]. This approach has been extensively documented in the series of Genotype-Tissue Expression (GTEx) project [12–14] in human. Currently, several eQTL studies have been conducted on farm animals, including cattle [15], pigs [16], sheep [17], and chicken [18]. For example, they observed that the production traits related to eQTLs also affect the expression of *MGST1* [19] and *SLC37A1* [20] in dairy cattle. However, eQTL analysis has not been conducted in ducks. Therefore, it is desirable to know whether eQTLs within GWAS signals contributed to duck economic traits.

In this study, we hypothesized that the colocalization of eQTL mapping and GWAS strategy could help identify essential candidate genes and causal DNA variants vital to growth and carcass traits. We integrated GWAS results of duck carcass and growth traits with the transcriptomes from seven tissues to prioritize genes and variants that influence duck economic traits through transcriptome. This work provided a duck *cis*-eQTL catalog consisting of 820 samples from seven tissues. The study results provide valuable resources for understanding the genetic effects on the transcriptome and also suggest the underlying molecular mechanisms of potentially causal functional variants in duck economic traits.

## Methods

### RNA-seq analysis

We downloaded 820 RNA-Seq datasets from Sequence Read Archive (SRA; https://www.ncbi.nlm.nih.gov/sra) and BIGD (https://bigd.big.ac.cn/bioproject/; Table S1). The adaptor and low-quality reads were trimmed and adaptor by Trimmomatic v0.39 [21]. The clean reads were mapped to the duck reference genome (ZJU1.0, https://www.ncbi.nlm.nih.gov/datasets/genome/GCF_015476345.1/) using STAR aligner [22]. To quantify gene expression, we used the mapped reads to calculate the gene expression levels using transcript per million (TPM) by StringTie [23] based on the annotation of RefSeq (GCF_015476345.1). A gene was considered expressed if it had a TPM threshold of ≥ 0.1 in at least 20% of the samples [15].

### Genotype imputation

PCR duplicates of STAR alignments were marked using MarkDuplicates in Picard (http://broadinstitute.github.io/picard/). We splited reads into exon segments and trimmed any sequences overhanging into the intronic regions using SplitNCigarReads modules of GATK v4.2.6.1 [24]. Then, we recalibrated base quality scores based on known genomic variants using BaseRecalibrator and ApplyBQSR modules of GATK. Then, we carried out joint-calling of all GVCF samples using the GenotypeGVCFs module of the GATK tool. The low-quality variants were filtered out using –filter-expression “FS > 30.0 & QD < 2.0”. We also check the variants consistency of different tissues from the same individual in PRJNA419583 study. The average of consistency was 99.6% (Table S2). The variants were imputed to DNA sequence variants level based on a multiple-breed reference panel consisting of 2215 Pekin ducks and 289 public individuals from Shaoxing ducks (*n* = 166) and wide mallards (*n* = 123) by Beagle 5.4 [25]. We removed variants with dosage *R*-squared (DR^2^) of less than 0.8, genotype call rates of less than 90%, minor allele frequency (MAF) of less than 0.05 or variants located outside of autosomes using PLINK v1.90 [26]. On average, we retained 1,112,867 autosomal variants with average DR^2^ of 0.92 for eQTL mapping.

### *Cis*-eQTL mapping

Gene expression values were normalized across samples using the inverse normal transformation. To account for population effects, we incorporated principal components (PCs) of genotype into the eQTL analyses. The number of PCs selected for the analysis was determined by testing their significance using EIGENSTRAT v6.1.4 (https://alkesgroup.broadinstitute.org/EIGENSOFT/). We ultimately decided to use the first three PCs for the eQTL analysis, as the test was not significant (*P*-value = 0.061) when more than three PCs were included (Table S3). Additionally, in order to address hidden batch effects and other sources of technical or biological variation, we estimated latent covariates for gene expression levels in each tissue using the Probabilistic Estimation of Expression Residuals (PEER) [27]. We retained 15 PEER confounding variables because the posterior variances of factor weights were nearly at their minimum values (Fig. S1). For blood tissue, we also conducted *cis*-eQTLs using 20 PEER confounding variables (https://github.com/WentaoCai/Duck_eQTL_results), we identified 1029 eGenes with 20 peer variables compared to 1040 eGenes using 15 peer variables. The number of overlapped eGenes was 952 (Fig. S2). We defined potential *cis*-eQTLs as variants located within a 1 Mb proximity ups or downstream of the transcription start sites (TSSs) of genes [28]. The *cis*-eQTLs mapping was conducted using a nominal *P*-value threshold that corresponded to FDR ≤ 0.05 for each gene by fastQTL [29]. The scripts of data analysis can be accessed on https://wentaocai.github.io/eQTL-analysis/. The gene lists were subjected to GO and KEGG analyses using DAVID with a significance threshold set at a *P*-value ≤ 0.05 [30]. We estimated the replication rate of *cis*-eQTL across different tissues using π_1_ statistic of the qvalue R package [31].

### GWAS and Colocalization

The phenotype of 50 growth and carcass traits were collected from 941 Pekin XMallard F2 individuals. The summary of phenotype records can be found in Table S4. The genotypes were conducted using resequencing technology on Illumina HiSeq X Ten, which was described in our previous study [8]. After mapping, call SNPs, and quality control, 13,064,619 variants for 941 individuals were obtained for GWAS. The phenotype was adjusted for covariates, including sex, feed room, and the first three PCs of genotypes. We used a linear mixed model for the association test of each SNP using the GCTA -mlma [32].

$${\varvec{y}}={\varvec{X}}{\varvec{\beta}}+{\varvec{u}}+{\varvec{\varepsilon}},$$

where ***y*** is adjusted phenotype; ***X*** is a vector of genotypes of a variant at the locus tested; ***β*** is the effect size of the variant; ***u*** is a vector of random polygenic effects ~ ***N*** (0, ${{\varvec{G}}{\varvec{\sigma}}}_{{\varvec{g}}}^{2}$), where ***G*** is genomic relationship matrix constructed from all variants; ***ε*** is a vector of residual errors.

We conducted the colocalization analysis of GWAS variants and eQTLs using Coloc R package [33]. The variants with *P*-value < 5 × 10^−5^ and their neighboring ± 50 Kb extracted from GWAS summaries of 50 growth and carcass traits were obtained for colocalization analysis [34]. GWAS signals and eQTLs were considered colocalizing if a posterior probability (H4) of the shared signal was > 0.4 [35].

## Results

### Transcriptome profile of duck primary tissues

**Fig. 1:**
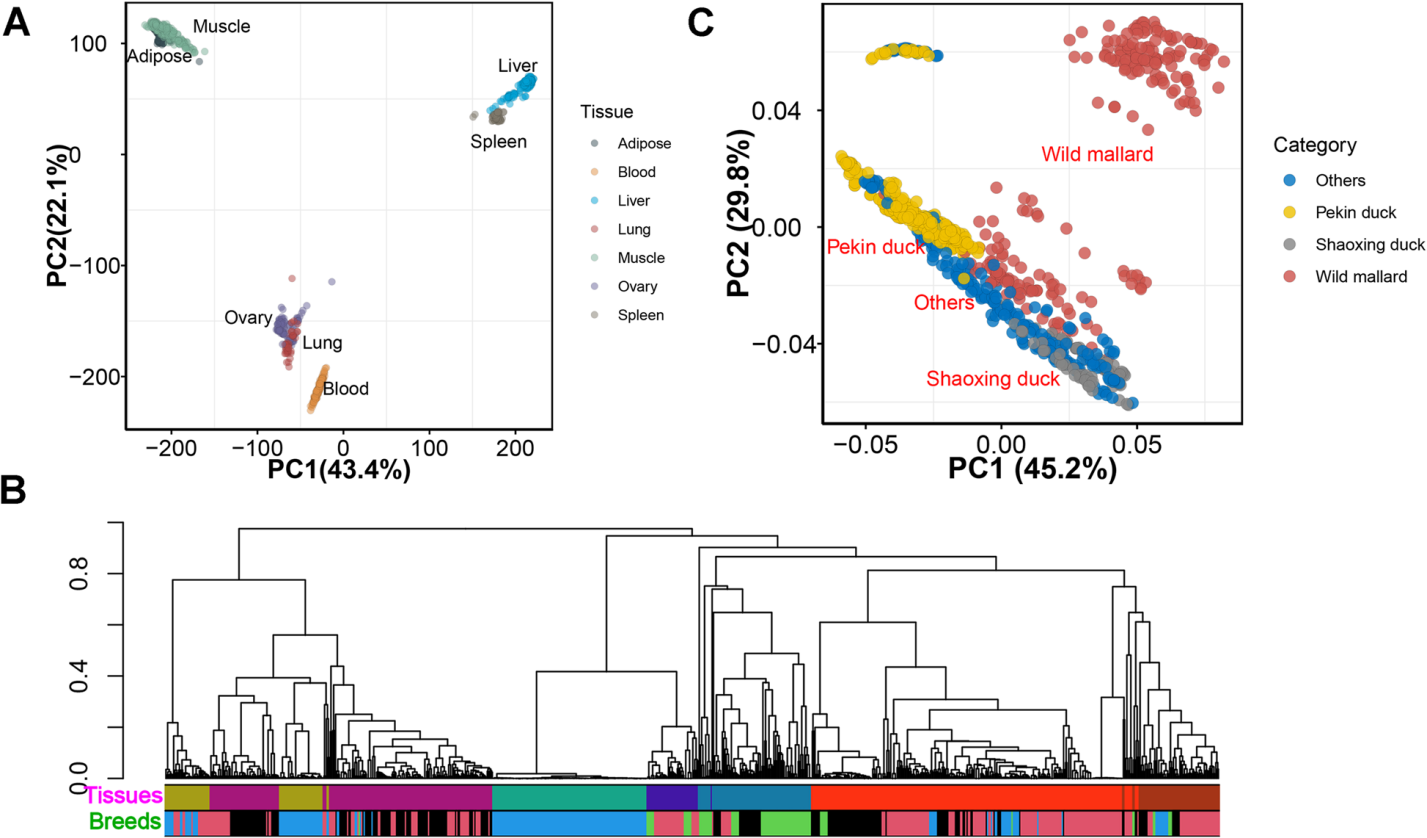
Principal component analysis (PCA) and hierarchical clustering of samples. **A** Sample clustering (*n* = 820) using PCA based on gene expression levels. **B** Hierarchical clustering of 820 samples using gene expression levels, sample clustering is affected by both tissues and breeds. **C** PCA of samples (*n* = 820) based on imputed genotypes

We obtained 22.5 billion clean reads from a total of 820 publicly available RNA-seq samples, including seven tissues (251 liver, 184 muscle, 120 blood, 71 adipose, 87 ovary, 66 spleen, and 41 lung) from 17 breeds (Table S1). Pekin ducks accounted for the largest proportion of samples (36.2%), indicative of their extensive economic significance worldwide. Approximately 88.9% of the total reads were successfully mapped to the reference genome (Table S1). Under the expression threshold of TPM ≥ 0.1 in at least 20% of the samples, 16,485 (65.7% of total genes) genes were expressed across tissues. Using PCA analysis, we can successfully distinguish samples according to different tissues and reconstruct the relationships between these tissues based on their expression levels (Fig. 1A). The expression profiles accurately reflected tissue types (Fig. 1B), which reaffirmed the high quality of these profiles and underscores their suitability for our subsequent analysis. We called variants from RNA-Seq samples and imputed each genotype using a reference population of 2,504 ducks. When applying PCA clustering to imputed genotypes, as anticipated, the samples exhibited clustering patterns corresponding to their respective breeds (Fig. 1C).

### Genetic effects on gene expression

**Fig. 2:**
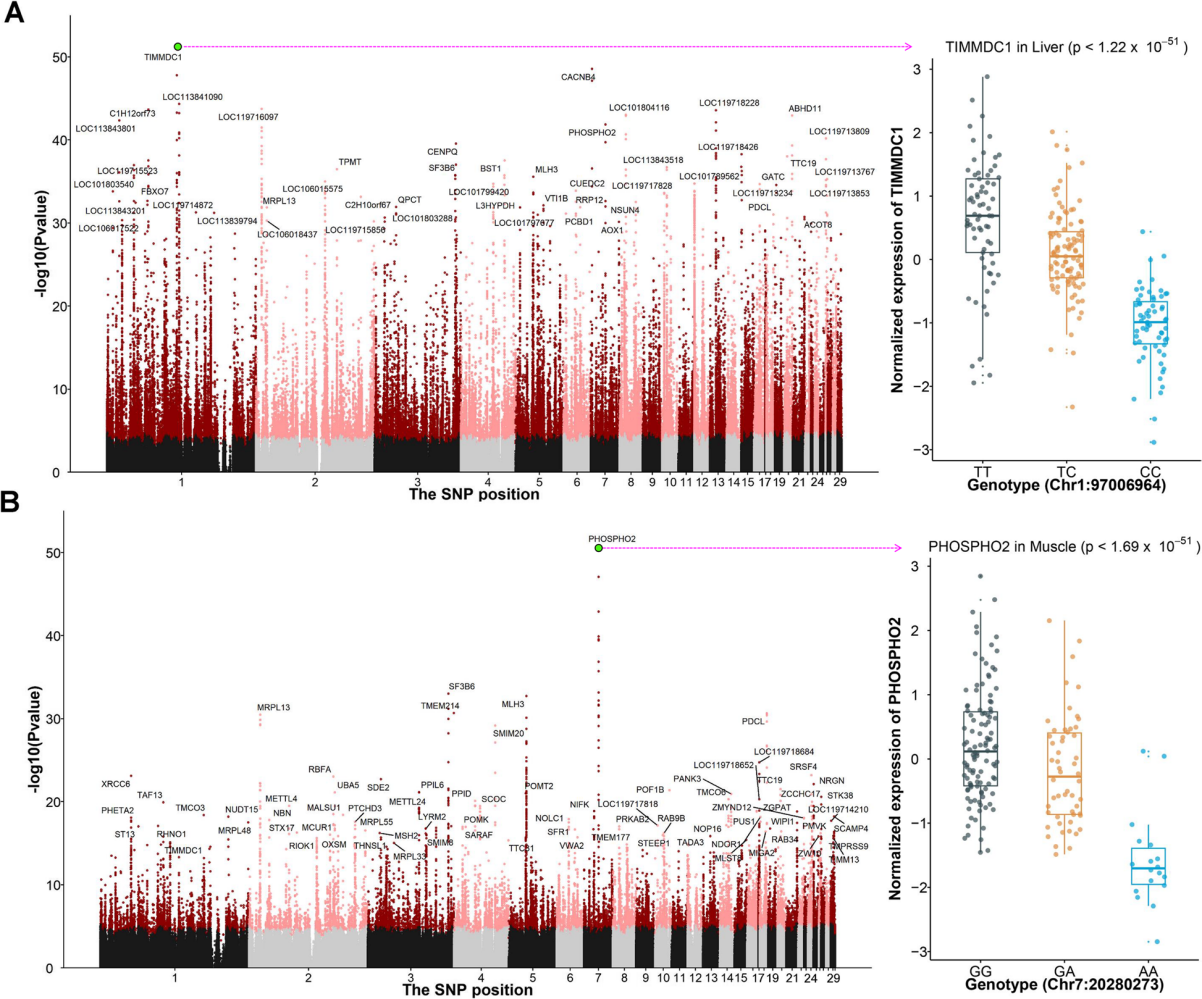
Manhattan plot of *cis*-eQTLs. **A** The Manhattan plot illustrates the nominal *P*-value (y-axis) for all *cis*-eQTLs in liver (left). Points colored dark red or pink represents significant *cis*-eQTLs, whereas those in black or gray were SNPs that did not reach a significant threshold. The expression of *TIMMDC1* with three genotypes in liver (right). **B** The Manhattan plot shows the nominal *P*-value (y-axis) for all *cis*-eQTLs in muscle (left). The expression of *PHOSPHO2* with three genotypes in muscle (right)

Considering all tissues, our analysis detected a total of 113,374 *cis*-eQTLs associated with 12,266 genes. These genes represent approximately 54.8% of all autosomally expressed genes. The numbers of eGene (gene with significant *cis*-eQTLs) for liver, muscle, blood, ovary, adipose, spleen, and lung were 7,301, 5,706, 1,040, 2,390, 989, 368, and 775, respectively (Table 1). The most notable *cis*-eQTLs detected in the liver were found to influence the expression of *TIMMDC1*, a gene crucial for the assembly of the membrane arm of mitochondrial Complex I (Fig. 2A). The most significant *cis*-eQTLs of muscle affected expression of *PHOSPHO2*, involved in dephosphorylation (Fig. 2B). The Manhattan plots of *cis*-eQTLs for other tissues are shown in Figs. S3, S4, S5, S6 and S7. The *cis*-eQTL results of seven tissues are accessed on https://github.com/WentaoCai/Duck_eQTL_results.

**Table 1:** The summary of *cis*-eQTL results in seven tissues. The genes, with an expression threshold of TPM (transcripts per million) ≥ 0.1 in at least 20% of samples on autosomes, account for the expressed genes. The eGene is a gene with significant *cis*-eQTLs. The eVariant is a genetic variant regulated at least one gene

Tissue	Sample size	Expressed genes	*Cis*-eGenes	*Cis*-eVariants	*Cis*-eQTL-gene pairs
Liver	251	14,537	7,301	73,267	104,594
Muscle	184	16,750	5,706	25,692	27,550
Blood	120	11,761	1,040	11,774	16,977
Ovary	87	20,503	2,390	7,437	7,847
Adipose	71	16,992	989	3,079	3,179
Spleen	66	15,744	368	1,365	1,368
Lung	41	17,137	775	5,109	5,452
Total	820	21,718^a^	12,266^a^	113,374^a^	159,240^a^

^a^Total item refers to the number of genes, variants or their pairs in the union across all seven tissues

### Character of *cis*-eQTLs

**Fig. 3:**
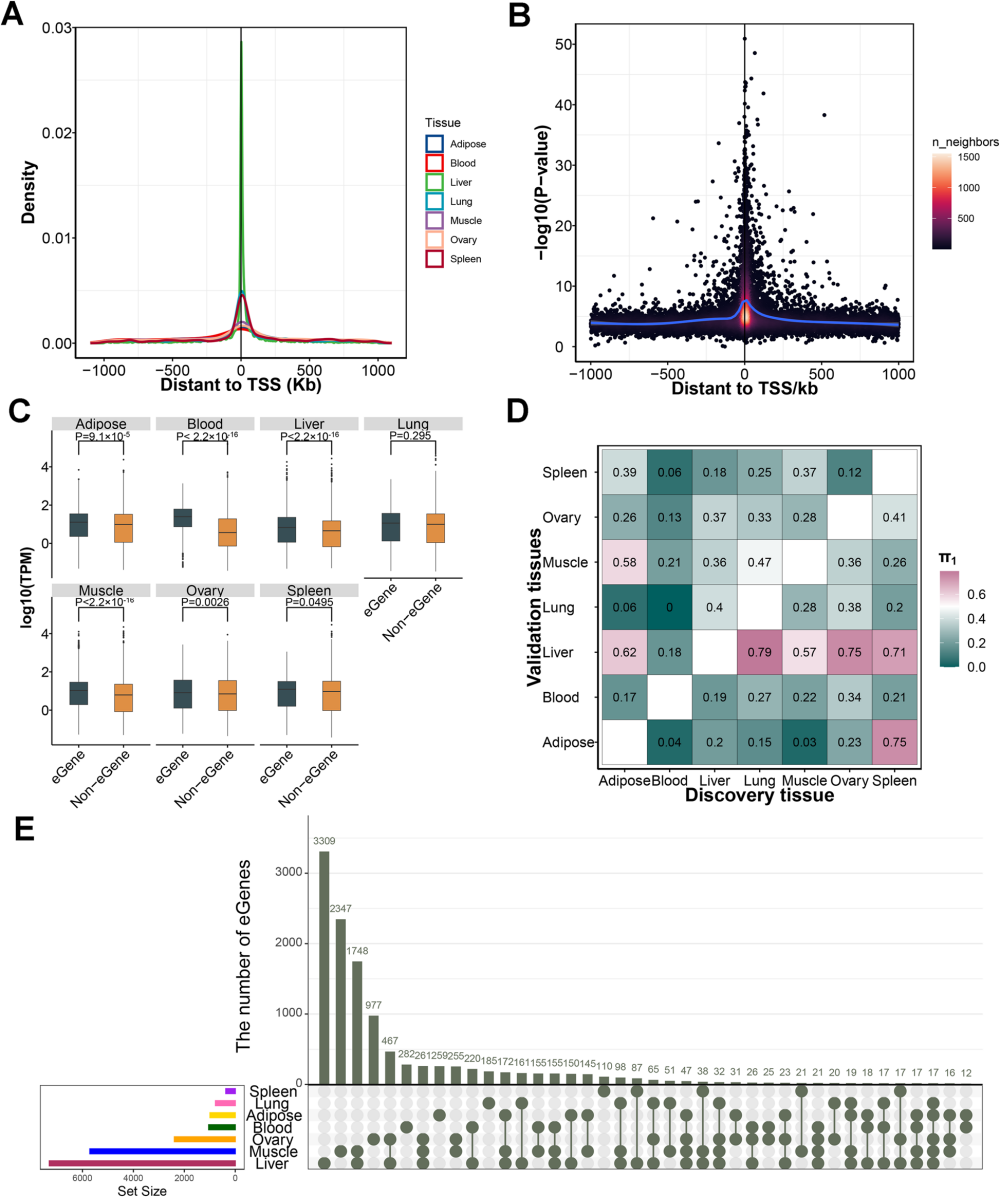
The characters of *cis*-eQTLs. **A** The density of top significant *cis*-eQTLs for each tested gene around TSS. **B** The *P*-value distribution of top significant *cis*-eQTLs for each tested gene in liver. **C** The expression of eGenes of *cis*-eQTL compared to non-eGenes. The significant *P*-value were calculated using Wilcoxon test. **D** Pairwise sharing patterns of *cis*-eQTL (π_1_ value) across tissues. **E** The overlaps of eGenes across tissues

Consistent with previous work in humans [36], most of the significant duck *cis*-eQTLs clustered around the TSS of target genes (Fig. 3A). An average of 45.3% of significant *cis*-eQTLs fell within 100 kb around the TSS. We found an enrichment of low *P* values closer to TSSs, showing that *cis*-eQTLs are more likely to be located within this distance (Fig. 3B and Fig. S8). *Cis*-eGenes of most tissues exhibited significantly higher expression levels compared to non-eGenes (Wilcoxon test, Bonferroni adjusted *P*-value < 0.05), with the exception of lung and spleen tissue (Fig. 3C). To assess the sharing patterns of *cis*-eQTLs between tissues, we computed the π_1_ statistics for each pair of tissues. We observed that *cis*-eQTLs in blood exhibited lower conservation across different tissues., while *cis*-eQTLs from any tissue exhibited a strong sharing pattern to liver tissue (Fig. 3D). We detected that any two tissues shared 39.1% of eGenes (Fig. 3E), and 10.7% of eVariants (Fig. S9). The 244 eGenes shared by at least four tissues were involved in basic metabolic pathways, such as nucleoplasm, mitochondrion, cytosol, and ATP binding (Table S5). Interestingly, we detected that the expression of *THNSL1*, *SF3B6*, *PPIL3*, *PHOSPHO2*, *LOC119713243,* and *LOC119717646* were affected by *cis*-eQTLs in almost all tissues except for spleen. On average, 10.7% of *cis*-eQTLs were involved in at least two eGenes.

### Colocalization of *cis*-eQTLs and GWAS loci

**Fig. 4:**
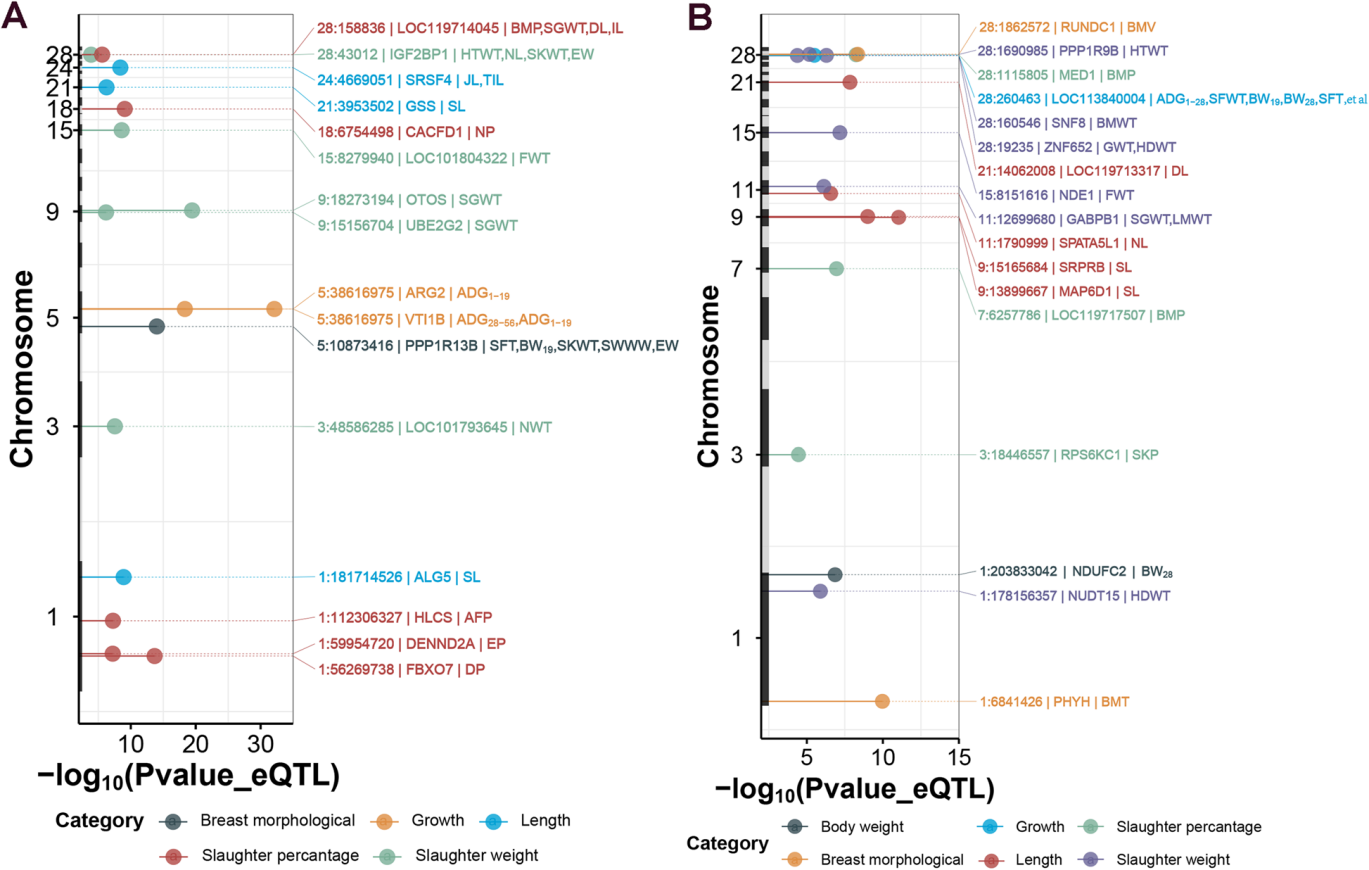
The colocalized results between *cis*-eQTLs and GWAS signals. **A** Manhattan plot illustrates the colocalization results (H4 > 0.4) between liver eQTLs and GWAS signals. The x-axis represents the *P*-value of lead eQTLs (points) across traits in liver. The right labels are colocalized SNP-gene-trait pairs. The different categories of tissues are distinguished by colors (**B**) Manhattan plot illustrates the colocalization results (H4 > 0.4) between muscle *cis*-eQTLs and GWAS signals

**Fig. 5:**
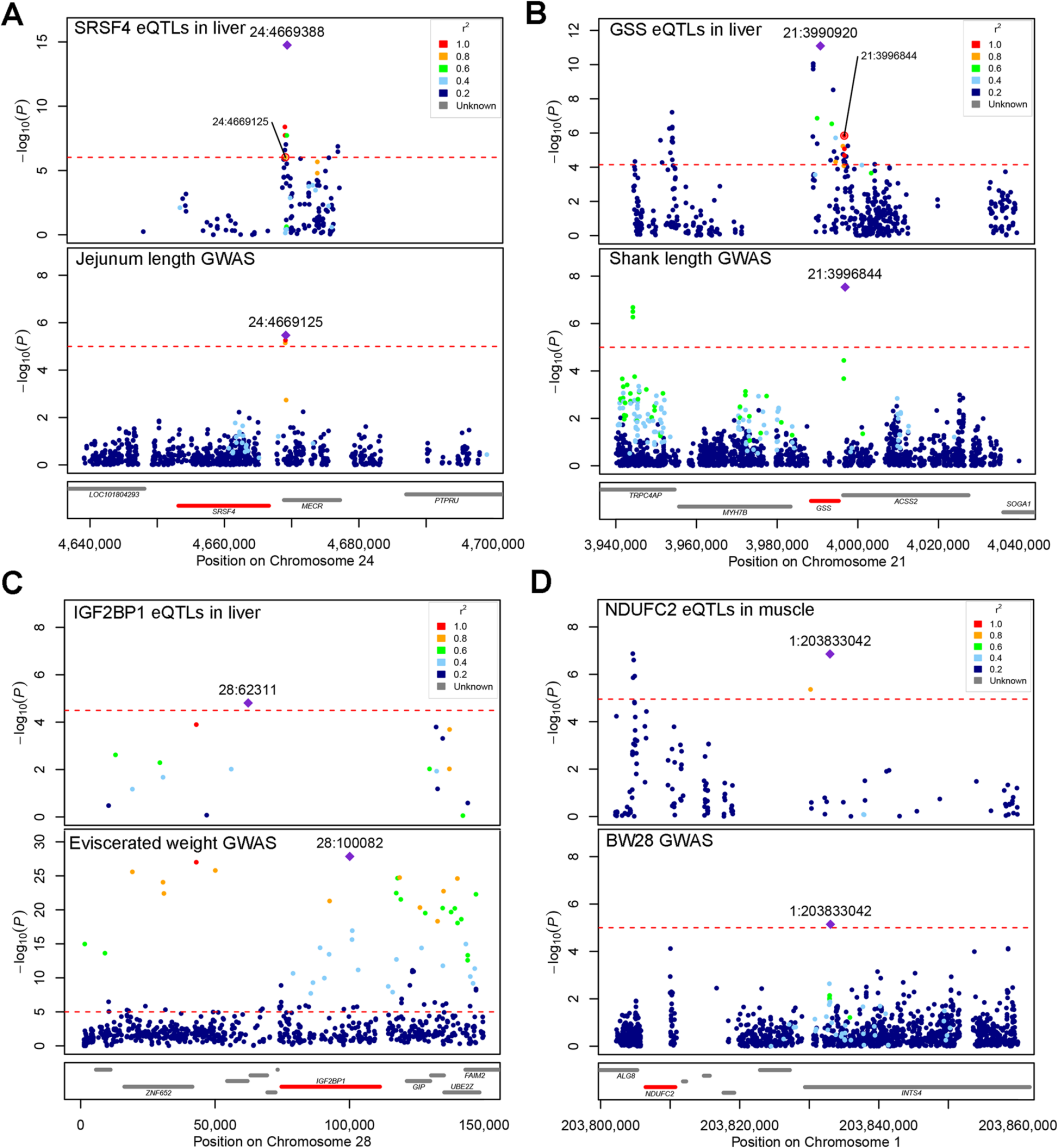
Examples of colocalized results between eQTLs and GWAS signals. **A** The colocalization between jejunum length GWAS and *SRSF4* eQTLs of liver. The colors of the variants are determined based on their linkage disequilibrium values with the most significant variant. **B** The colocalization between shank length GWAS and *GSS* eQTLs of liver. **C** The colocalization between eviscerated weight GWAS and *IGFBP1* eQTLs of liver. **D** The colocalization between BW_28_ (body weight at 28 days) GWAS and *NDUFC2* eQTLs of muscle

We conducted GWAS between genomic variants and 50 growth and carcass traits in F_2_ ducks (Table S4). We identified a total of 2,136 unique variants that showed significant association (*P* < 5 × 10^–8^) with 47 agronomic traits, of which, 1,039 variants were associated with at least two traits (Table S6). We obtained the variants with *P*-value < 5 × 10^−5^ and their neighboring ± 50 Kb for colocalization analysis. We observed that the eQTLs for 49 eGenes in seven tissues were colocalized with 41 traits, resulting in a total of 94 tissue-gene-trait pairs (Fig. 4, Table 2, Fig. S10, and Table S5). In liver, we detected that eQTLs of *SRSF4* (serine and arginine rich splicing factor 4) were colocalized with GWAS signals of jejunum length and total intestine length (Fig. 5A). The top GWAS signal of jejunum length significantly affecting the expression of *SRSF4.* The eQTLs of *GSS* (glutathione synthetase) were colocalized with GWAS signals of shank length (Fig. 5B). There was a strong LD (*r*^*2*^ = 1) between the top eQTLs signal and the top GWAS signal. The top GWAS signal of shank length is also significantly associated with the expression of *GSS.* Previously, we detected the variants located *IGF2BP1* gene affecting the carcass traits and body size [8]. Here, we confirmed these GWAS signals can affect the phenotype of eviscerated weight, heart weight, neck length, and skeleton weight through the change of *IGF2BP1* expression (Fig. 5C and Table S7).

**Table 2:** The colocalized results of eGene-trait pairs. Colocalization analyses were conducted between *cis*-eQTLs and GWAS traits

Tissue	The colocalization pairs of eGene and trait
Adipose	*ELF3* → BW_56_; *LOC101795547* → BMWT; *LOC101795662* → HDP; *LOC119713076* → EW, SFWT, CWT, BW_19_, SFT; *LOC119714005* → GWT; *TACC2* → LWT; *VTI1B* → ADG_1-19_, ADG_28-56_
Blood	*CEP19* → BW_56_; *DNAJC13* → LGMP; *GPR155* → BMP; *RUNDC1* → IL
Liver	*ALG5* → SL; *ARG2* → ADG_1-19_; *CACFD1* → NP; *DENND2A* → EP; *FBXO7* → DP; *GSS* → SL; *HLCS* → AFP; *IGF2BP1* → HTWT, NL, SKWT, EW; *LOC101793645* → NWT; *LOC101804322* → FWT; *LOC119714045* → BMP, SGWT, DL, IL; *OTOS* → SGWT; *PPP1R13B* → SFT, BW_19_, SKWT, SWWW, EW; *SRSF4* → JL, TIL; *UBE2G2* → SGWT; *VTI1B* → ADG_1-19_, ADG_28-56_
Lung	*LOC113840671* → GWT; *LOC119714045* → BMP; *LOC119714053* → NP
Muscle	*GABPB1* → SGWT, LMWT; *LOC113840004* → ADG_1-28_, SFWT, BW_28_, BW_19_, SFT, NWT, CWT, EW, LMWT, SWWW, SKWT, BW_56_, BMP, TIL, JL, ADG_1-56_, BW_1_, BMV, DL, IL; *LOC119713317* → DL; *LOC119717507* → BMP; *MAP6D1* → SL; *MED1* → BMP; *NDE1* → FWT; *NDUFC2* → BW_28_; *NUDT15* → HDWT; *PHYH* → BMT; *PPP1R9B* → HTWT; *RPS6KC1* → SKP; *RUNDC1* → BMV; *SNF8* → BMWT; *SPATA5L1* → NL; *SRPRB* → SL; *ZNF652* → GWT, HDWT
Ovary	*GTF2H5* → FP; *IPPK* → NP; *LOC119718243* → SFWT, AFWT; *RIT1* → FWT
Spleen	*PLA2G4A* → BMV

The arrow symbol denotes *cis*-regulated genes that mediate the association between genetic variants and traits. *ADG*_*1-19*_ Average daily gain from 1 to 19 days, *ADG*_*1-28*_ Average daily gain from 1 to 28 days, *ADG*_*1-56*_ Average daily gain from 1 to 56 days, *ADG*_*28-56*_ Average daily gain from 28 to 56 days, *AFP* Abdominal fat percentage, *AFWT* Abdominal fat weight, *BMP* Breast muscle percentage, *BMT* Breast muscle thickness, *BMV* Breast muscle volume, *BMWT* Breast muscle weight, *BW*_*1*_ Body weight at 1 day, *BW*_*19*_ Body weight at 19 days, *BW*_*28*_ Body weight at 28 days, *BW*_*56*_ Body weight at 56 days, *CWT* Carcass weight, *DL* Duodenum length, *DP* Dressed percentage, *EP* Eviscerated percentage, *EW* Eviscerated weight, *FP* Feet percentage, *FWT* Feet weight, *GWT* Gizzard weight, *HDP* Head percentage, *HDWT* Head weight, *HTWT* Heart weight, *IL* Ileum length, *JL* Jejunum length, *LGMP* Leg muscle percentage, *LMWT* Leg muscle weight, *LWT* Liver weight, *NL* Neck length, *NP* Neck percentage, *NWT* Neck weight, *SFT* Skin and fat thickness, *SFWT* Skin subcutaneous fat weight, *SGWT* Swing weight, *SKP* Skeleton percentage, *SKWT* Skeleton weight, *SL* Shank length, *SWWW* Skeleton without wings weight, *TIL* Total intestine length

In muscle tissue, the eQTLs of *NDUFC2* (NADH: ubiquinone oxidoreductase subunit C2) were colocalized with BW_28_ (body weight at 28 days; Fig. 5D). The top significant GWAS signals for BW_28_ and the top significant eQTLs of *NDUFC2* were the same. It appeared that there were two independent loci in eQTL signals of liver *GSS* and muscle *NDUFC2* (Fig. 5B and D). To confirm whether the eQTL signals were derived from the same causative variant, we repeated *cis*-eQTL mapping using the top eQTL signal of *GSS* or *NDUFC2* as a covariate. No significant signals were detected using this conditional analysis (Fig. S11), indicating the eQTL signals of liver *GSS* and muscle *NDUFC2* arise from one causative variant. The eQTLs of *LOC113840004* were colocalized with 20 traits, implying this uncharacterized gene would be an important functional gene in ducks. In other tissues, we observed the eQTLs of *EIF3* (E74 like ETS transcription factor 3) in adipose were associated with the GWAS of BW_56_ body weight at 56 days (Table S7). The eQTLs of *RUNDC1* (RUN domain containing) in blood were detected colocalized with the GWAS of ileum length. The eQTLs of *IPPK* (inositol-pentakisphosphate 2-kinase) in ovary were detected colocalized with the GWAS of neck weight percentage. Overall, the colocalization analysis enhanced our ability to identify potentially causal genes and gain a deeper understanding of the genetic underpinnings of complex traits in ducks.

## Discussion

In this study, we created a comprehensive catalog of eQTLs across multiple tissues, further expanding the list of candidate genes and potential variants affecting important agronomic traits in ducks.

Consistent with other species [36, 37], a high density of signals was observed near the TSSs of their respective genes. The majority of eQTLs appear to be tissue-specific, suggesting intricate and distinct genetic mechanisms governing gene expression across different tissues. The functional annotation of tissue-shared eGenes revealed that genetic variants more prominently and frequently impact genes related to immune and metabolic functions, which is consistent with previous findings in human [38] and cattle [37]. Similar to cattle [37], these *cis*-eGenes in duck were found to exhibit higher expression levels than non-eGenes in most tissues.

The colocalization analysis between GWAS signals and *cis*-eQTLs in the seven tissues facilitates the identification of causal genes for association signals that were previously unresolved. Several GWAS signals were observed to colocalize with *cis*-eQTLs. *SRSF4* belongs to the family of Arginine-Serine-rich (SR) proteins, which play a crucial role in constitutive splicing and also regulate alternative splicing [39]. Like other SRSFs, *SRSF4* shuttles between the nucleus and cytoplasm, and mediate mRNA regulation, including export, stability, and translation [40, 41]. *SRSF4* is associated with human colon adenocarcinomas [42]. *SRSF4* has a relatively high expression in the liver, stomach, and intestine [43]. The upstream variants of *SRSF4* have significantly regulated the expression of *SRSF4* in the liver for both cattle [15] and pigs [16]. GSS is responsible for catalyzing the condensation of gamma-glutamylcysteine and glycine to produce glutathione (GSH) [44]. GSH is essential for a multitude of processes, such as safeguarding cells from oxidative damage, facilitating amino acid transport, detoxifying foreign compounds, preserving protein sulfhydryl groups in a reduced state, and serving as a cofactor for several enzymes [45]. The mutations of GSS are associated with body height in humans [46, 47]. The length of the shank directly determines a poultry's height. The top GWAS signal of shank length is also significantly associated with the expression of *GSS*, which implies that *GSS* could be a candidate gene for the shank length and body height of the duck. *IGF2BP1* belongs to the insulin-like growth factor 2 mRNA-binding protein family and plays a role in RNA transport, cell proliferation, differentiation, and metabolism by regulating the mRNA localization, stability, and translation of specific target genes. [48, 49]. *IGF2BP1* is a well-known candidate gene for body size in ducks [8], chickens [50], and humans [51]. Here, we confirmed the variants of *IGF2BP1* can regulate the duck body size by changing the *IGF2BP1* expression in liver.

*NDUFC2* is a subunit of the mitochondrial membrane respiratory chain NADH dehydrogenase (Complex I) [52]. A deficiency in *NDUFC2* results in cellular-level mitochondrial dysfunction and increased oxidative stress [53]. *NDUFC2* appears to be downregulated in the skeletal muscle cells of individuals with insulin resistance and is linked to insulin secretion in vivo [54, 55]. The mutations of *NDUFC2* are associated with body mass index [56]. Both BW_28_ GWAS and *NDUFC2* eQTLs shared the same of top significant variant, implying *NDUFC2* may act as a candidate gene for duck body weight. *ELF3*, an important member of the E74-like transcription factor family, involves in inflammatory response [57] and adipogenic differentiation [58]. Leptin is a potent pro-inflammatory and pro-catabolic factor, and its downstream actions are mediated by *ELF3* [59]. The mutations near *ELF3* were associated with body mass index in humans [60]. The eQTLs of *ELF3* in adipose were associated with BW_56_. *RUNDC1* is an inhibitor of the tumor suppressor p53 [61], which can negatively modulate autophagy by blocking fusion between autophagosomes and lysosomes [62]. The eQTLs of *RUNDC1* in blood were associated with ileum length. We can hypothesize that *RUNDC1* is a candidate gene for ileum length. These results are crucial for gaining a deeper understanding of the molecular mechanisms underlying specific traits by considering gene expression and the functional characteristics of genes associated with these traits.

Although our study released thousands of eGenes by analyzing public RNA-seq data, there are still some limitations. For example, some tissues have limited sample sizes, resulting in the detection of a small number of eGenes in several tissues, such as spleen, lung, and adipose. The potential biases in variant detection, population stratification resulting from the inclusion of data from multiple breeds, and confounding factors stemming from diverse experimental designs may not have been completely resolved when using publicly available RNA-seq data. In addition, the variety and sample size of our multiple-breed reference panel for genotype imputation remain limited, which may introduce biases in the genotype results. This study serves as a pioneering investigation into the field of duck eQTLs. The summary statistics of duck eQTLs are still unavailable, which impedes a more detailed investigation of colocalized signals and thus an evaluation of possible pleiotropic effects. Our analysis of GWAS signals within the context of duck eQTLs serves as a preliminary foundation for future investigations in this field.

## Conclusions

The interpretation of genetic mechanisms underlying complex traits based on the molecular phenotype of primary tissues in ducks was rare. Through the integration of eQTL and GWAS data, we have constructed a molecular QTL map in ducks, which aids in unraveling genetic association signals by identifying candidate genes, such as *SRSF4*, *GSS*, *IGF2BP1, NDUFC2, ELF3*, and *RUNDC1*. Moreover, these newly identified *cis*-eQTLs and candidate genes will enhance the accuracy of genomic prediction and contribute to the benefit of genetic improvement programs in duck breeding.

### Supplementary Information

**Additional file 1: Fig. S1.** Characterization of PEER factors. Factor weight variance as a function of PEER factors computed up to 30 factors for each of seven tissues. Factor weight variances become small for tissues when the number of inferred hidden PEER factors reaches 15.

**Additional file 2: Fig. S2.** The number of overlapping eGenes identified using 15 and 20 peer variables in blood tissues.

**Additional file 3: Fig. S3.** The distribution of *cis*-eQTLs in blood.

**Additional file 4: Fig. S4.** The distribution of *cis*-eQTLs in ovary.

**Additional file 5: Fig. S5.** The distribution of *cis*-eQTLs in adipose.

**Additional file 6: Fig. S6.** The distribution of *cis*-eQTLs in spleen.

**Additional file 7: Fig. S7.** The distribution of *cis*-eQTLs in lung.

**Additional file 8: Fig. S8.** The *P*-value distribution of top significant *cis*-eQTLs for each tested gene in muscle, blood, ovary, adipose, spleen and lung.

**Additional file 9: Fig. S9.** The number of eVariants overlaps between tissues.

**Additional file 10: Fig. S10.** The colocalized results between *cis*-eQTLs and GWAS signals in other five tissues. Manhattan plot illustrates the colocalization results (H4 > 0.4) between *cis*-eQTLs and GWAS signals. The x-axis is the *P*-value of lead eQTLs (points) across trait categories (colors). The right labels are colocalized SNP-gene-trait pairs.

**Additional file 11: Fig. S11. **The *cis*-QTL signals of liver *GSS* and muscle *NDUFC2* using conditional analysis. (A) The *cis*-QTL signals of liver *GSS* using the top eQTL signal 21:3990920 as a covariate. (B) The *cis*-QTL signals of muscle *NDUFC2* using the top eQTL signal 21:203833042 as a covariate.

**Additional file 12: Table S1.** The information for 820 RNA-seq samples.

**Additional file 13: Table S2.** The consistency of SNPs calling using different RNA-seq samples from the same individuals.

**Additional file 14: Table S3.** Result of principal component significance test for genotype.

**Additional file 15: Table S4.** The summary of GWAS results for 50 agronomic traits.

**Additional file 16: Table S5.** GO and KEEG annotation of 244 eGenes shared by at least four tissues.

**Additional file 17: **Table S6**.** The GWAS results of significant signals with *P* < 5 × 10^–8^ for 47 traits.

**Additional file 18: Table S7.** The results of GWAS-eQTL colocalization by Coloc in seven tissues.

## Data Availability

All eQTL results are available on GitHub with the identifier https://github.com/WentaoCai/Duck_eQTL_results. All the genotype data of duck F_2_ population have been deposited in the Sequence Read Archive (https://www.ncbi.nlm.nih.gov/sra) with the accession numbers PRJNA471401 and PRJNA450892. Other results were provided in the Additional file. The scripts used for data processing and analyses are available on the website through the following link, https://wentaocai.github.io/eQTL-analysis/.

## Abbreviations

eQTLs: Expression quantitative trait loci
FDR: False discovery rate
GO: Gene Ontology
GTEx: The Genotype-Tissue Expression
GWAS: Genome-wide associated studies
KEGG: Kyoto Encyclopedia of Genes and Genomes
LD: Linkage disequilibrium
PCs: Principal components
PCA: Principal component analysis
PEER: Probabilistic estimation of expression residuals
SNPs: Single nucleotide polymorphisms
TPM: Transcripts per million
TSS: Transcription start site
