# Supplementary figures and images for: Cis-eQTLs in seven duck tissues identify novel candidate genes for growth and carcass traits
Source: BMC Genomics. 2024 Apr 30;25:429. doi: 10.1186/s12864-024-10338-7 (PMC11061949; doi:10.1186/s12864-024-10338-7)

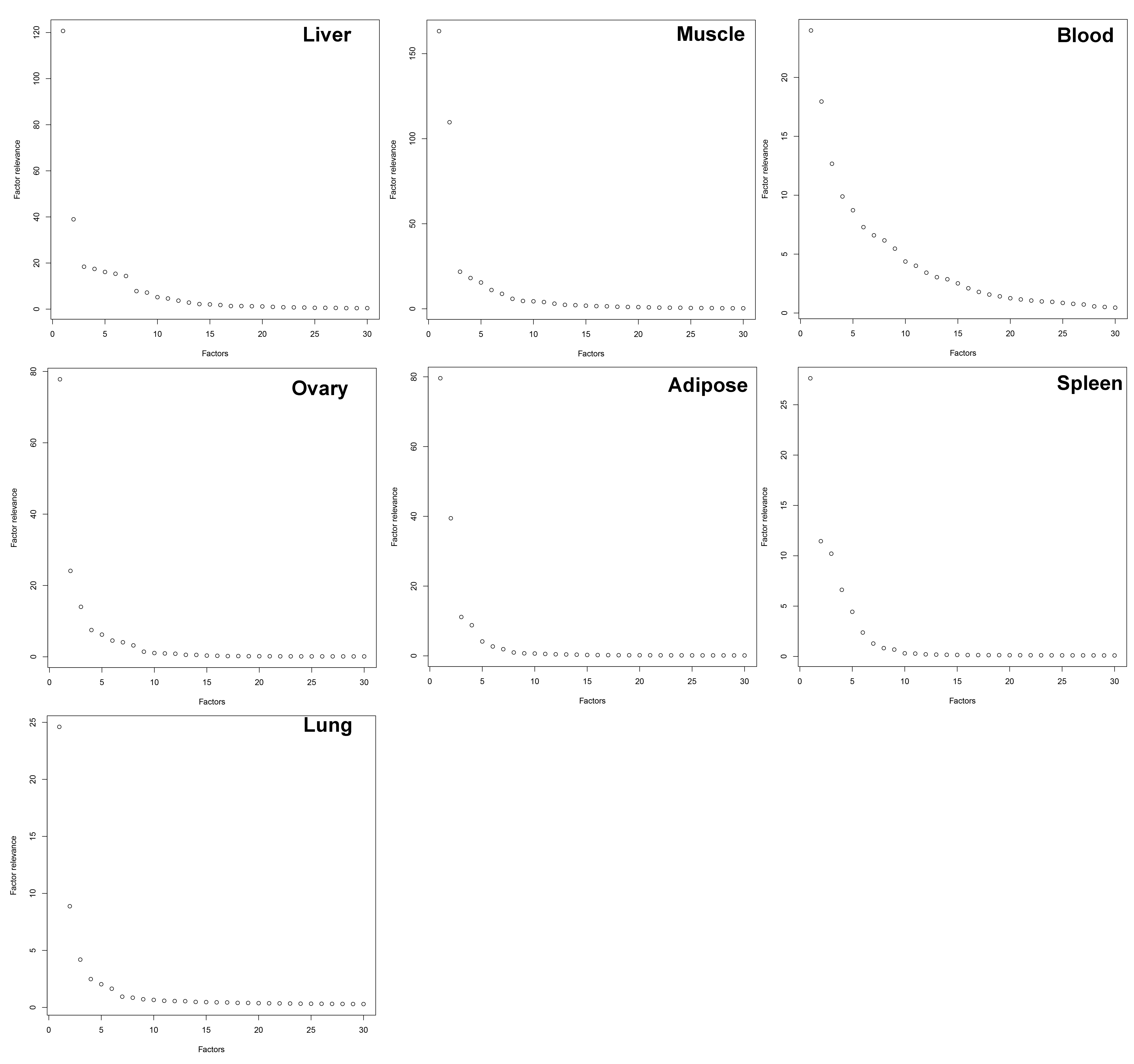

Supplement: Supplementary file 1 — Additional file 1: Fig. S1. Characterization of PEER factors. Factor weight variance as a function of PEER factors computed up to 30 factors for each of seven tissues. Factor weight variances become small for tissues when the number of inferred hidden PEER factors reaches 15. [file 12864_2024_10338_MOESM1_ESM.tif]

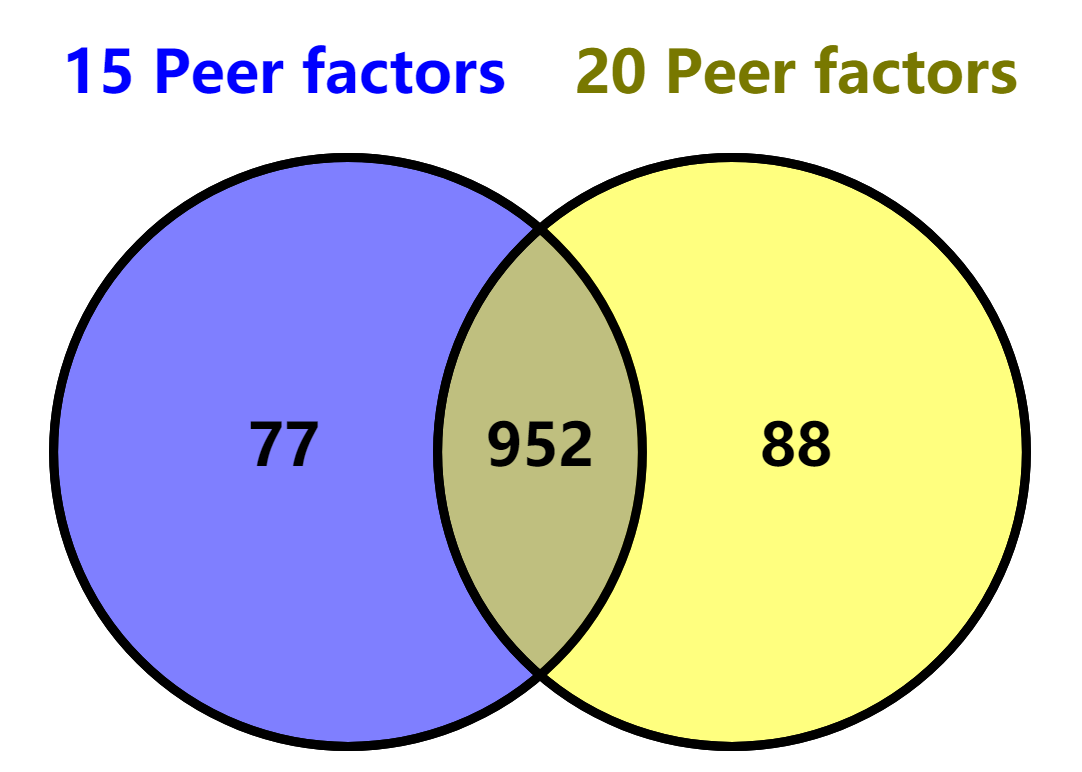

Supplement: Supplementary file 2 — Additional file 2: Fig. S2. The number of overlapping eGenes identified using 15 and 20 peer variables in blood tissues. [file 12864_2024_10338_MOESM2_ESM.tif]

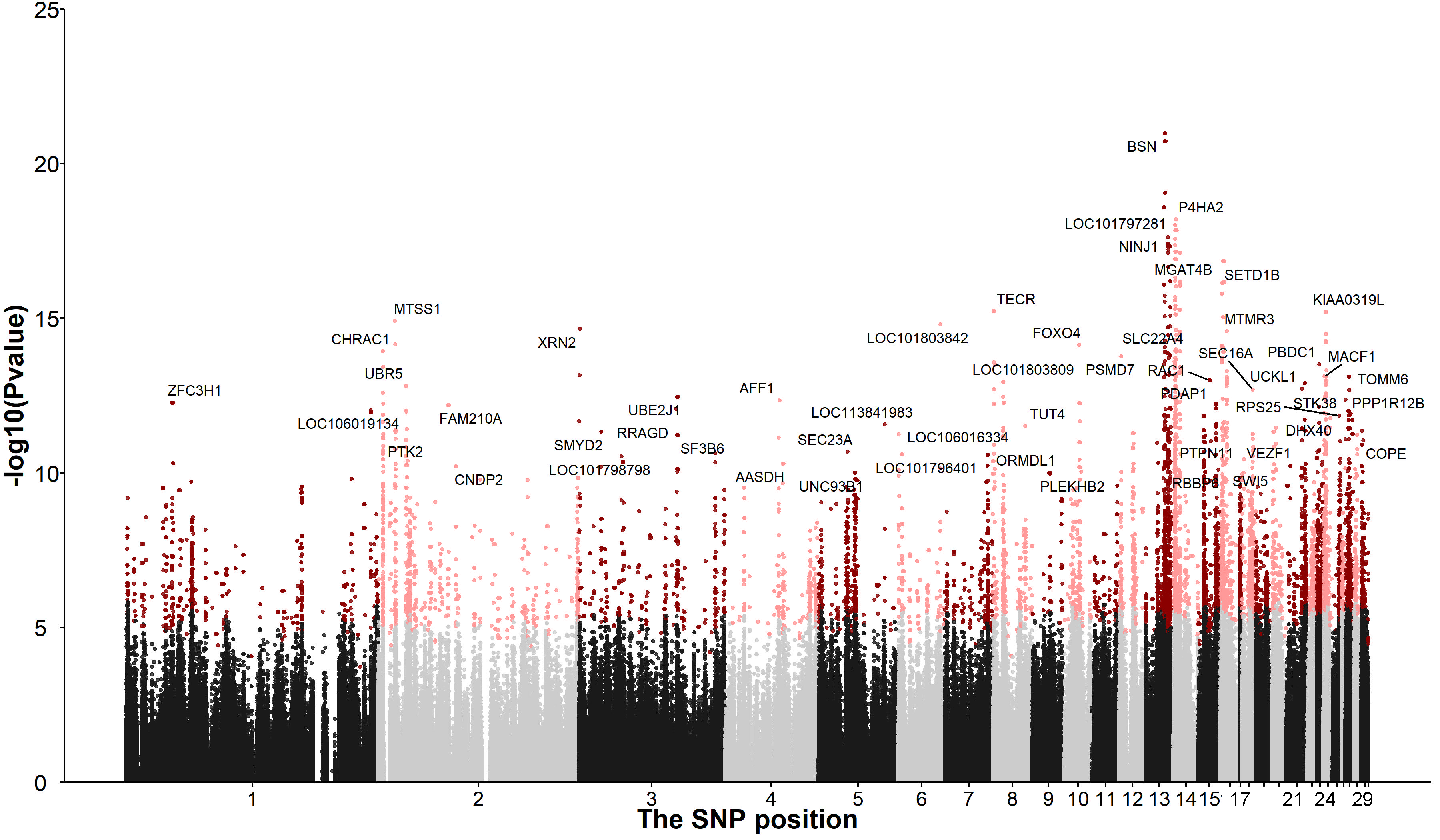

Supplement: Supplementary file 3 — Additional file 3: Fig. S3. The distribution of cis-eQTLs in blood. [file 12864_2024_10338_MOESM3_ESM.tiff]

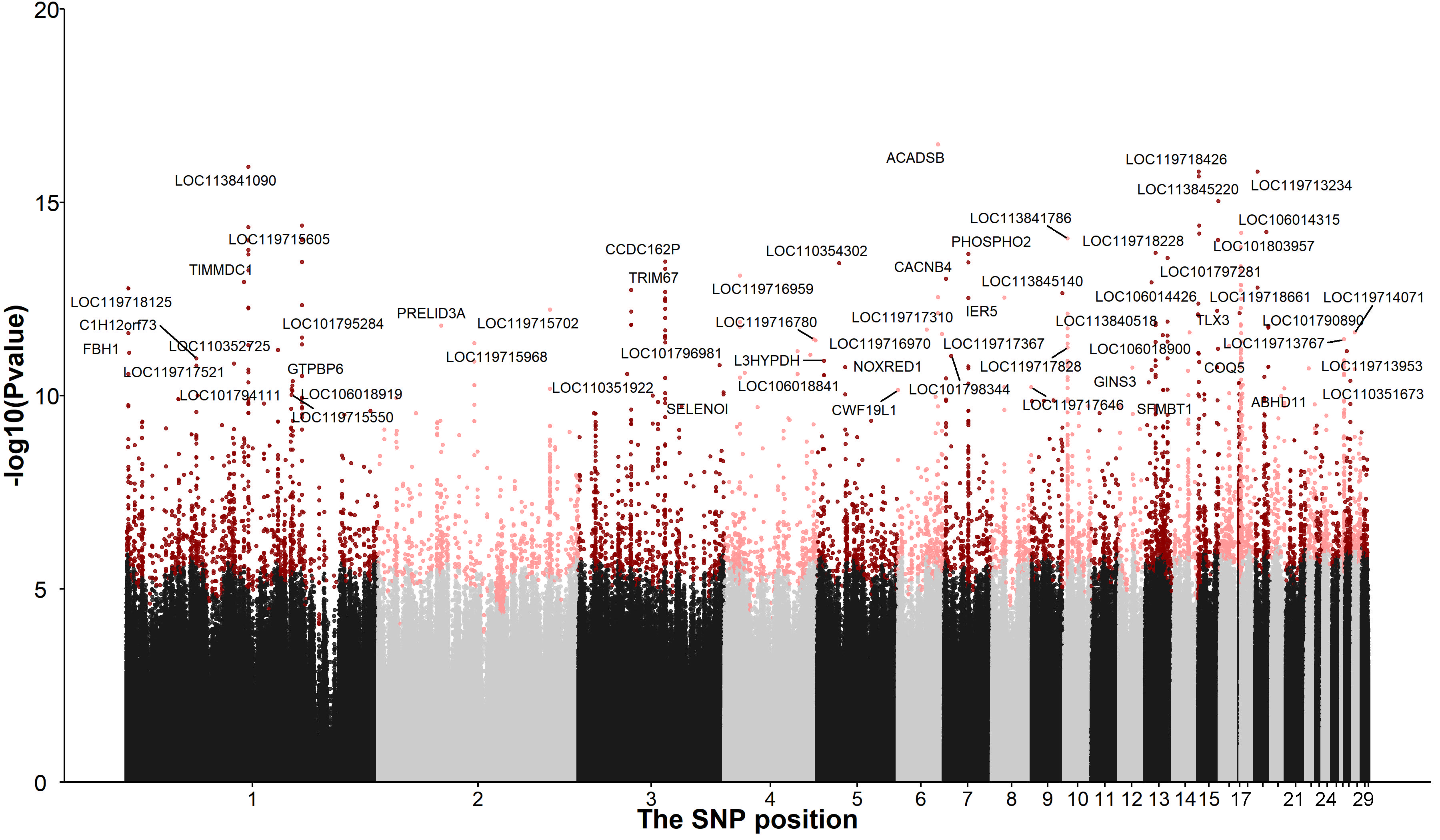

Supplement: Supplementary file 4 — Additional file 4: Fig. S4. The distribution of cis-eQTLs in ovary. [file 12864_2024_10338_MOESM4_ESM.tiff]

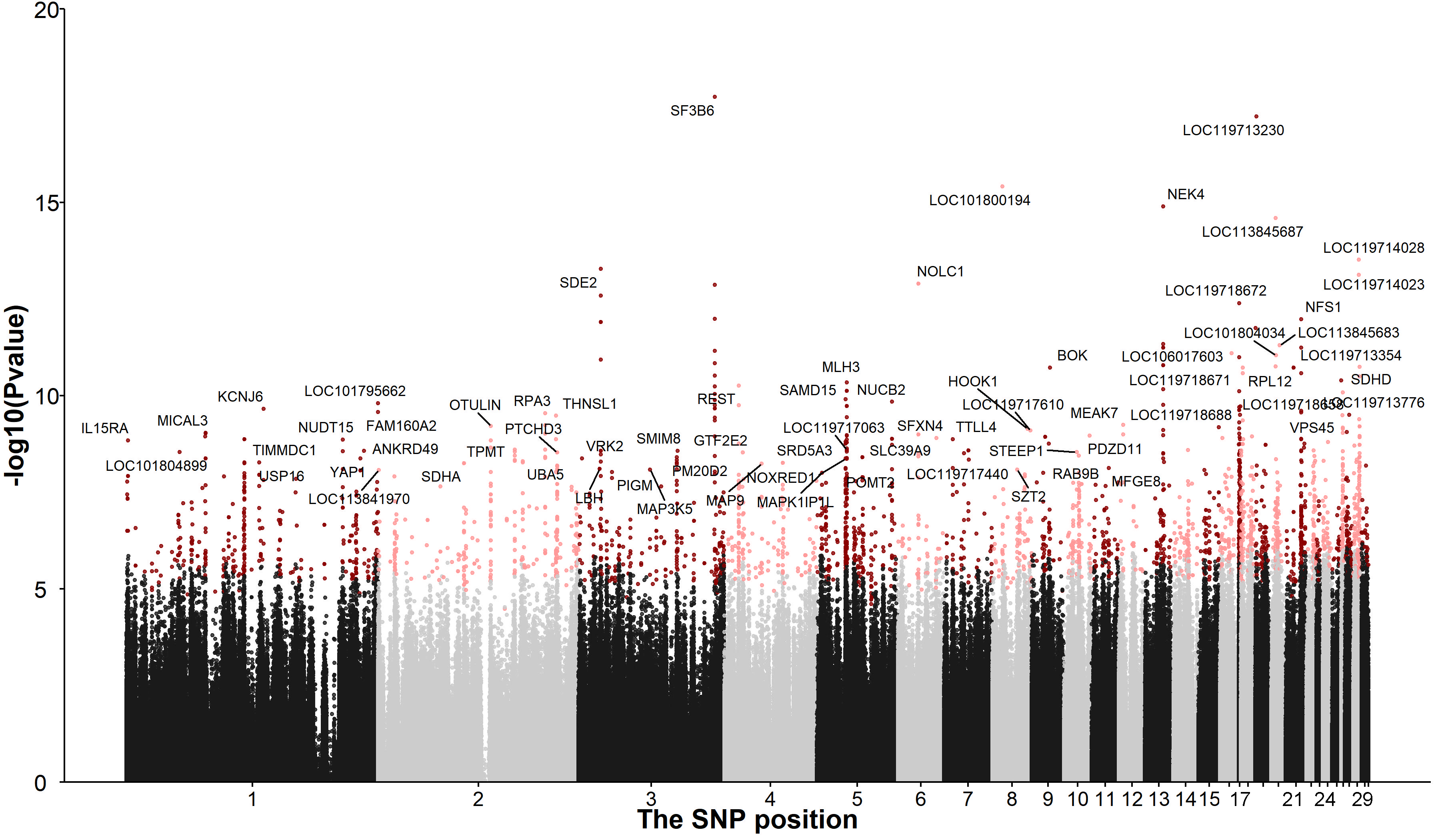

Supplement: Supplementary file 5 — Additional file 5: Fig. S5. The distribution of cis-eQTLs in adipose. [file 12864_2024_10338_MOESM5_ESM.tiff]

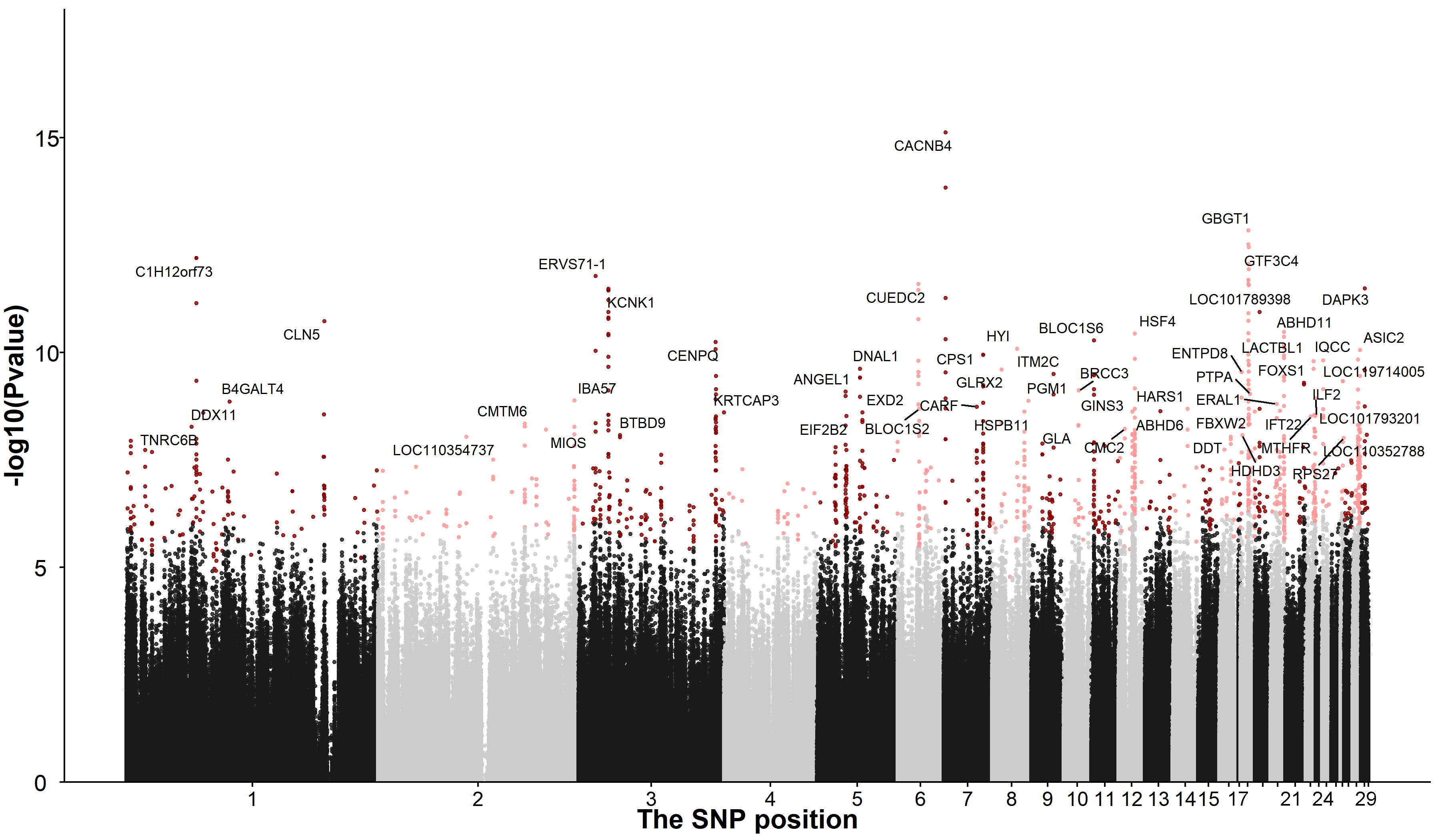

Supplement: Supplementary file 6 — Additional file 6: Fig. S6. The distribution of cis-eQTLs in spleen. [file 12864_2024_10338_MOESM6_ESM.tiff]

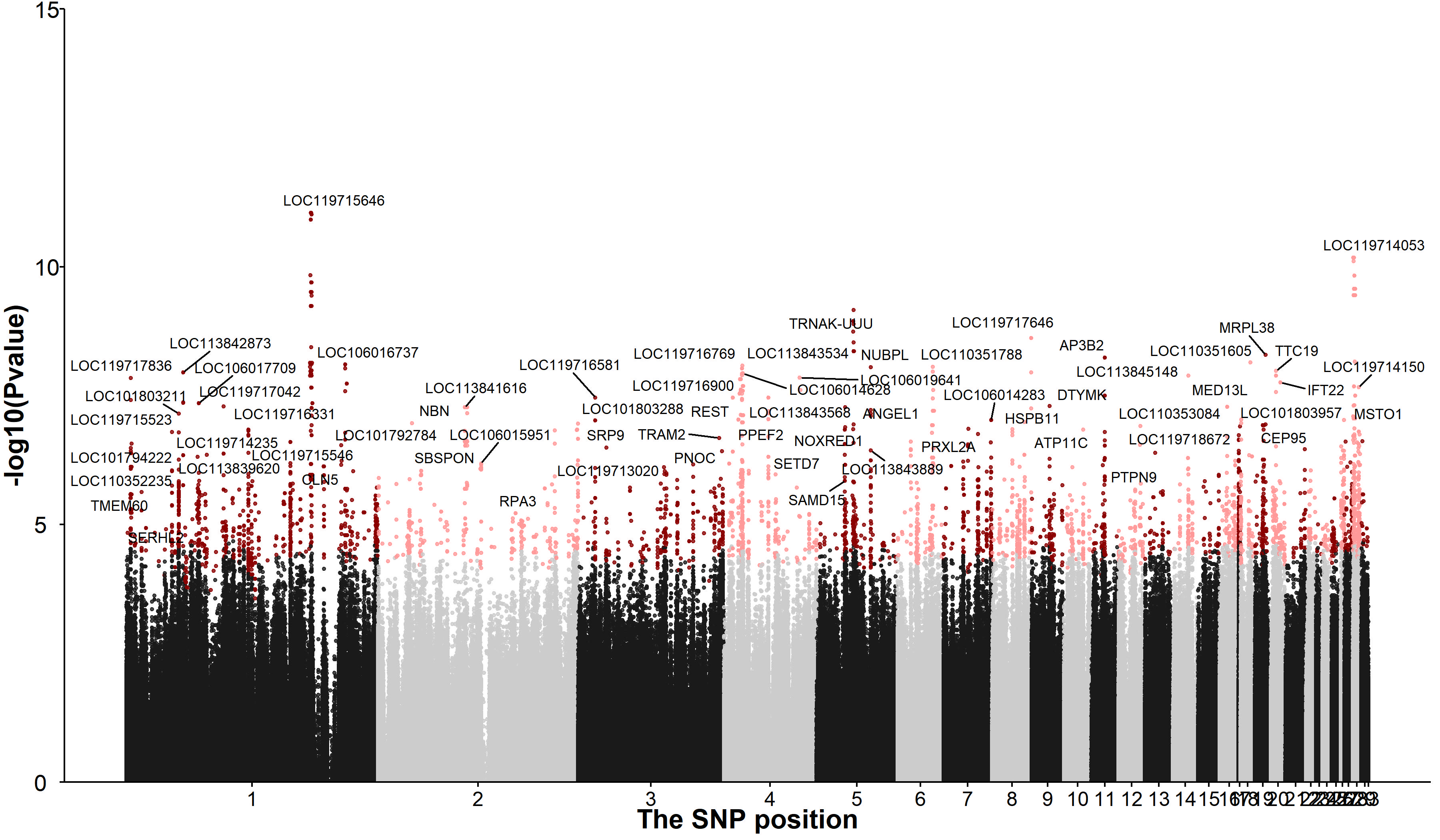

Supplement: Supplementary file 7 — Additional file 7: Fig. S7. The distribution of cis-eQTLs in lung. [file 12864_2024_10338_MOESM7_ESM.tiff]

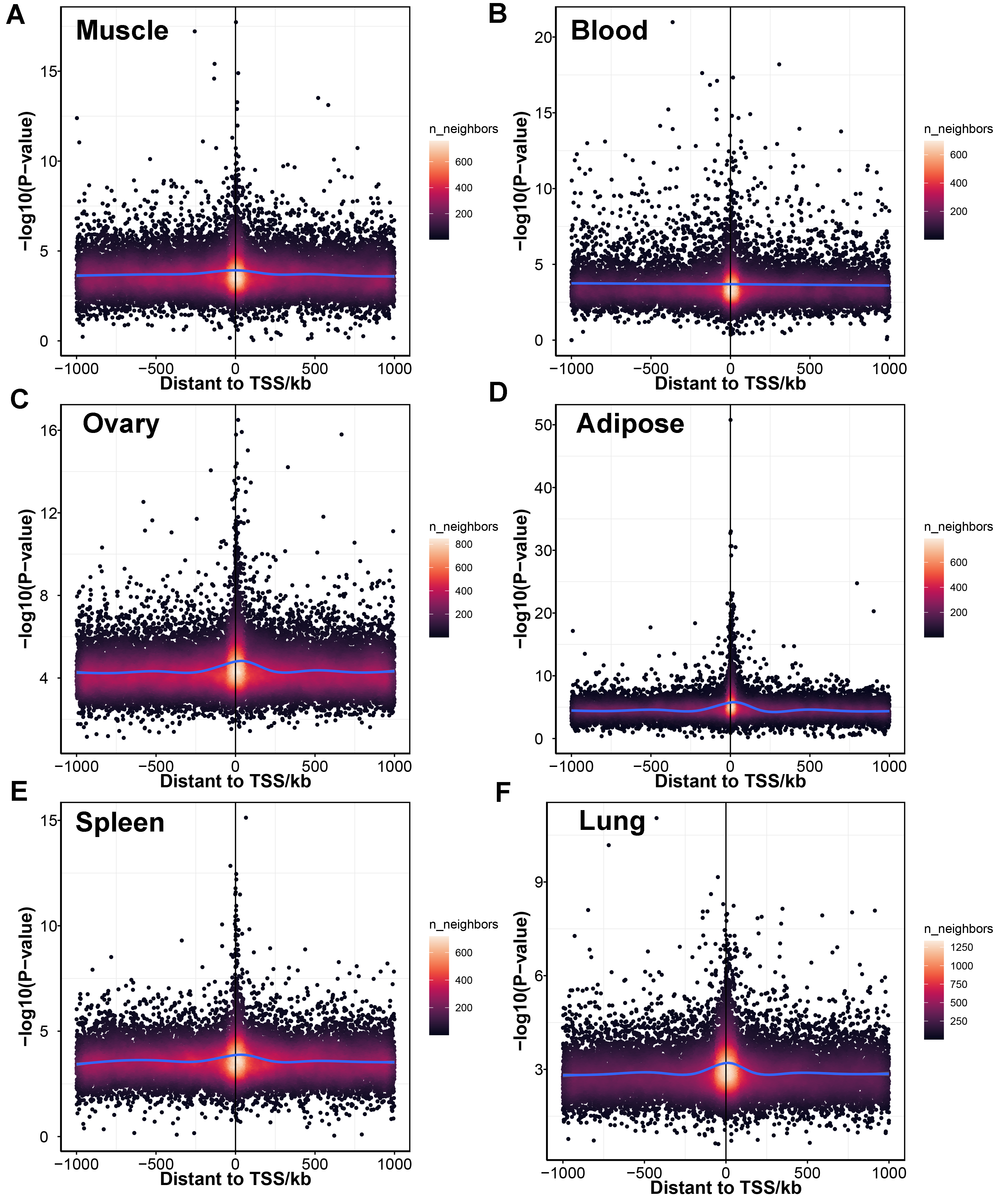

Supplement: Supplementary file 8 — Additional file 8: Fig. S8. The P-value distribution of top significant cis-eQTLs for each tested gene in muscle, blood, ovary, adipose, spleen and lung. [file 12864_2024_10338_MOESM8_ESM.tif]

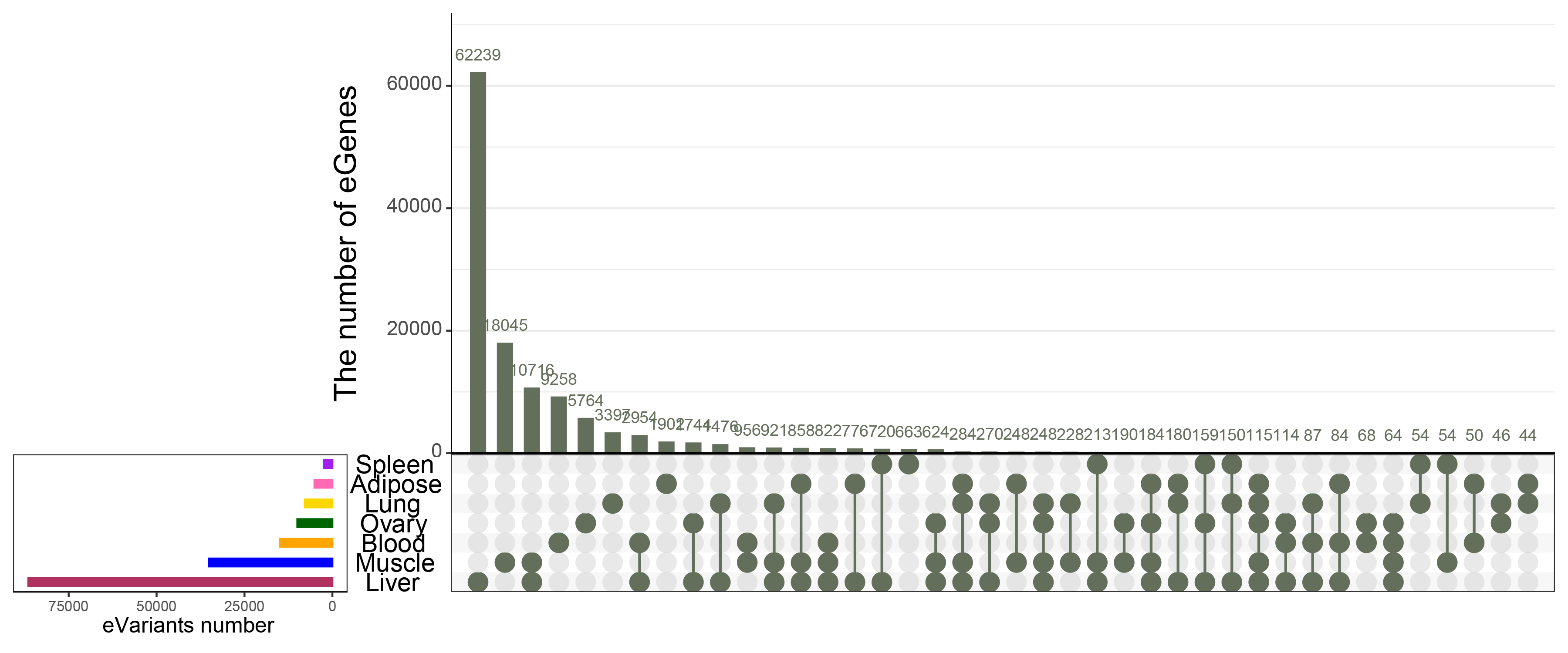

Supplement: Supplementary file 9 — Additional file 9: Fig. S9. The number of eVariants overlaps between tissues. [file 12864_2024_10338_MOESM9_ESM.tif]

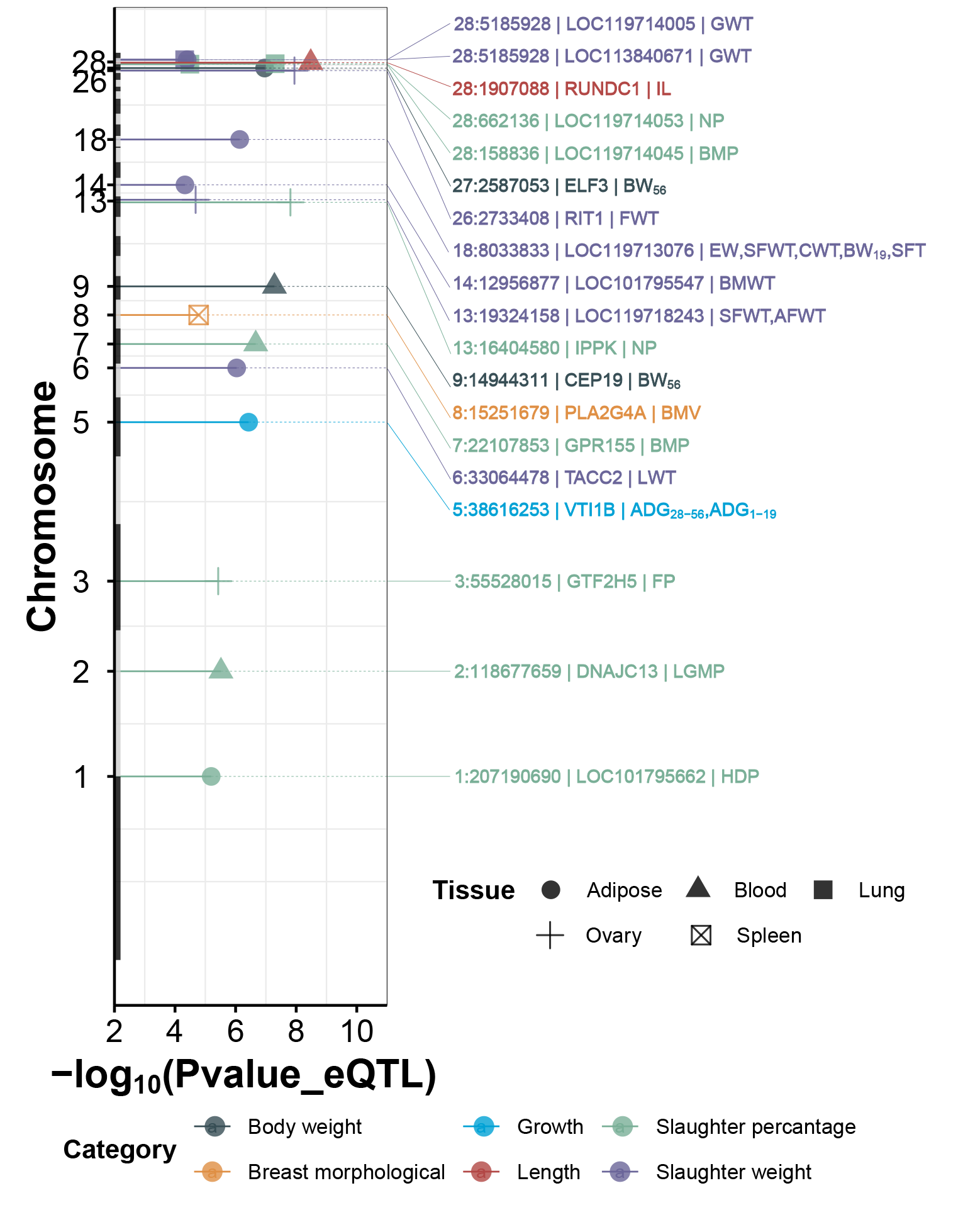

Supplement: Supplementary file 10 — Additional file 10: Fig. S10. The colocalized results between cis-eQTLs and GWAS signals in other five tissues. Manhattan plot illustrates the colocalization results (H4 > 0.4) between cis-eQTLs and GWAS signals. The x-axis is the P-value of lead eQTLs (points) across trait categories (colors). The right labels are colocalized SNP-gene-trait pairs. [file 12864_2024_10338_MOESM10_ESM.tif]

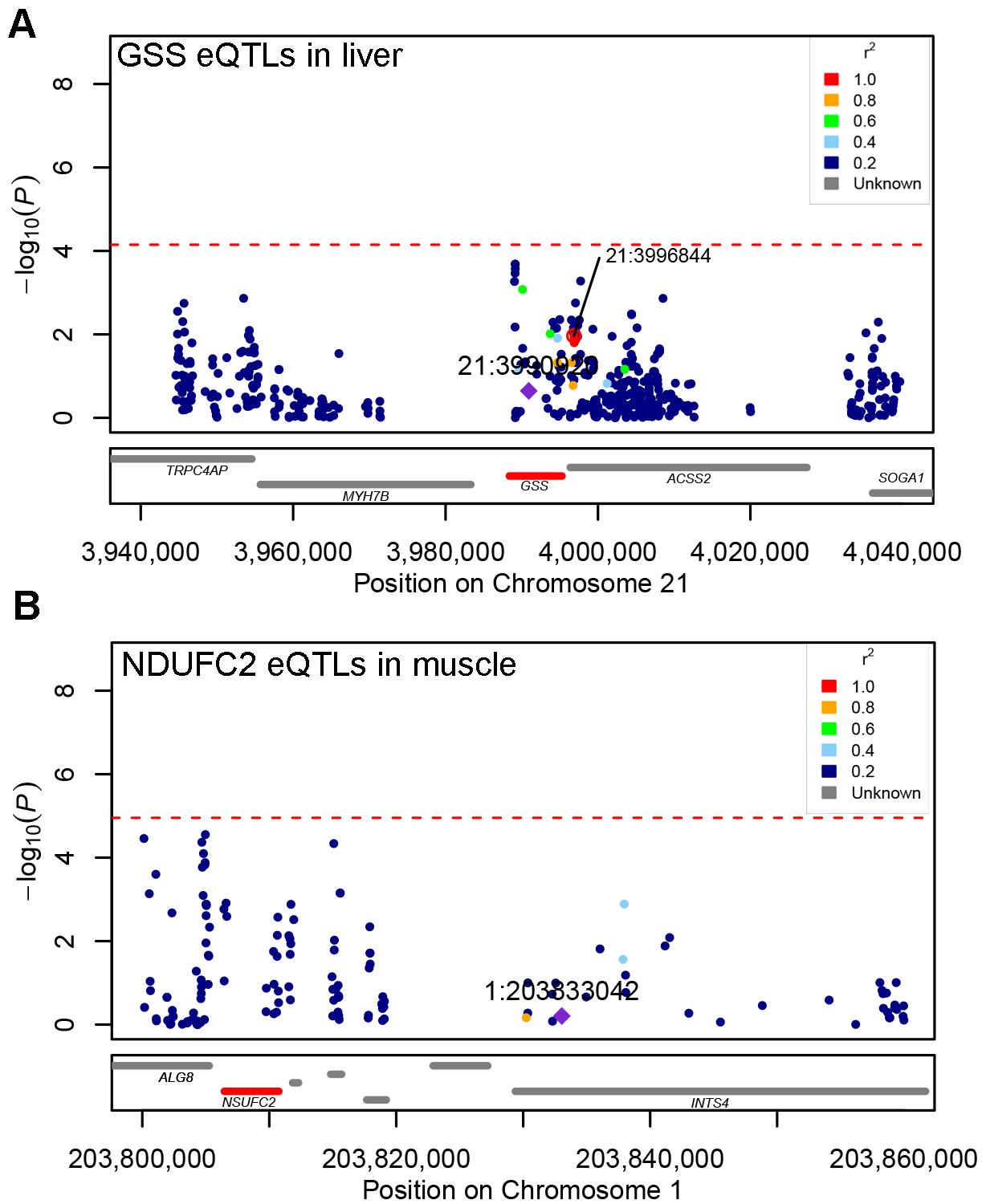

Supplement: Supplementary file 11 — Additional file 11: Fig. S11. The cis-QTL signals of liver GSS and muscle NDUFC2 using conditional analysis. (A) The cis-QTL signals of liver GSS using the top eQTL signal 21:3990920 as a covariate. (B) The cis-QTL signals of muscle NDUFC2 using the top eQTL signal 21:203833042 as a covariate. [file 12864_2024_10338_MOESM11_ESM.tif]
